# Modelization of the Current and Future Habitat Suitability of *Rhododendron ferrugineum* Using Potential Snow Accumulation

**DOI:** 10.1371/journal.pone.0147324

**Published:** 2016-01-29

**Authors:** Benjamin Komac, Pere Esteban, Laura Trapero, Roger Caritg

**Affiliations:** 1 Centre d’Estudis de la Neu i la Muntanya d’Andorra, Institut d'Estudis Andorrans (CENMA - IEA), Avinguda Rocafort 21–23, AD600 Sant Julià de Lòria, Principality of Andorra; 2 Departament de Geografia Física i Anàlisi Geogràfica Regional, Facultat de Geografia i Història, Universitat de Barcelona, Carrer de Montalegre 6–8, 08001 Barcelona, Spain; Lakehead University, CANADA

## Abstract

Mountain areas are particularly sensitive to climate change. Species distribution models predict important extinctions in these areas whose magnitude will depend on a number of different factors. Here we examine the possible impact of climate change on the *Rhododendron ferrugineum* (alpenrose) niche in Andorra (Pyrenees). This species currently occupies 14.6 km^2^ of this country and relies on the protection afforded by snow cover in winter. We used high-resolution climatic data, potential snow accumulation and a combined forecasting method to obtain the realized niche model of this species. Subsequently, we used data from the high-resolution Scampei project climate change projection for the A2, A1B and B1 scenarios to model its future realized niche model. The modelization performed well when predicting the species’s distribution, which improved when we considered the potential snow accumulation, the most important variable influencing its distribution. We thus obtained a potential extent of about 70.7 km^2^ or 15.1% of the country. We observed an elevation lag distribution between the current and potential distribution of the species, probably due to its slow colonization rate and the small-scale survey of seedlings. Under the three climatic scenarios, the realized niche model of the species will be reduced by 37.9–70.1 km^2^ by the end of the century and it will become confined to what are today screes and rocky hillside habitats. The particular effects of climate change on seedling establishment, as well as on the species’ plasticity and sensitivity in the event of a reduction of the snow cover, could worsen these predictions.

## Introduction

Mountainous regions are biodiversity-rich areas [1], principally due to the pronounced topographical and climatic gradients that exist over short distances [2]. Their endemic diversity is highly vulnerable to climate change [3] since small changes can have serious consequences [4]. Thus, the conservation of their biodiversity has become an important challenge in these regions [5]. Species distribution models (SDMs) [6] based on the evaluation of species’ bioclimatic niches are often used to explore plant and animal distributions because they can be applied to conservation tasks in mountain areas where climatic gradients are particularly appropriate for assessing species’ responses to climate [7, 8].

The recent use of SDMs in climate change analyses has shown that there is an important risk of species extinction throughout the planet; the impact of climate change on species suitability, and on abundance and extinctions rates will determine the magnitude of future change [9, 10]. Although an increase in species diversity is to be expected in alpine and subnival vegetation belts as a consequence of upward shifts by vegetation (see [11]; and reference therein), climate change still represents an important extinction risk for species and will lead to the loss of suitable habitats and the reduction of dispersal opportunities [12, 13]. In the Pyrenees, for example, the predicted responses to climate change in mountain areas include the upward migration of *Pinus uncinata*, the most abundant conifer in this cordillera [14], an upstream colonization by the freshwater fish *Barbus barbus* [15], an important decline in climatically suitable habitat for the lycaenid butterfly *Lycaena helle* [16] and a little or no shrinkage in the distribution of the at-risk endemic Pyrenean desman *Galemys pyrenaicus* [17]. These differing predicted responses by animal and plant species to climate change are directly related to each species’ sensitivity to climate change and so a general consideration of species-specific ecological niches is liable to reveal useful trends [18].

In this work, we examine the possible impact of climate change on the habitat suitability of the shrub *Rhododendron ferrugineum*, the alpenrose, in Andorra (Eastern Pyrenees). This species’ particularity is its dependence on snow in winter and spring [19, 20]. Snow cover is imperative for the winter survival of many subalpine species because it allays extreme temperatures that may exceed plant frost tolerance, helps avoid plant desiccation and protects evergreen plants from excessive irradiation (see [20, 2]; and references therein). In the case of *R*. *ferrugineum*, winter snow cover prevents plant species leaves, buds and roots from freezing, but also permits more efficient photosynthesis, as plant recovery is greater when freezing does not occur [20]. For example, important frost damage is observed in the leaves and flowers of a *R*. *ferrugineum* population when frosts occur during spring—and even in mid-July—and affect, above all, the plant’s flowers (A. Pornon *com*. *pers*.). The conservation of the alpenrose habitat is important as a contribution to the stabilization of insecure eroded mountain soils that harbour characteristic vascular plants and alpine fungi. This is also a key habitat for the threatened black grouse (*Tetrao tetrix*) [21] and is one of the heathland habitat types included in the Alpine and Boreal heaths of the European Habitats Directive [22]. In certain French mountain areas, an important decline in snow cover duration is expected to occur by the end of the present century due to climate change [23], which could have negative consequences for the survival of this habitat.

One of the most interesting aspects of SDMs for this study is their ability to assess the effect of climate change on a species realized niche that is mainly dependent on snow cover. Nevertheless, the attention paid to date to snow cover in SDMs is relatively poor and models tend to focus on the consequences of changes in the duration of the plant-growing season [24] or of the snowpack [25]. Here, we use the high resolution of the Climatological Atlas of Andorra [26] to compute the potential snow accumulation [27] in order to obtain a more accurate model of the distribution of *R*. *ferrugineum* in Andorra and a more realistic framework for climate change scenario modelling.

Over the last decade, with the increased use of SDMs in ecological species niche modelling, differing terminology regarding the definition of niche species has been employed due to disagreements or diverse points of view [28]. This is why, following [28], we use the term ‘realized niche model’ in this study to determine the habitat suitability map for *R*. *ferrugineum* in Andorra. The species realized niche obtained using topographic and climatic variables reveals certain tendencies regarding a species’ sensitivity to climate change [29]. Thus, we use potential snow accumulation here to obtain highly accurate models of the bioclimatic suitability of *R*. *ferrugineum* in Andorra and to acquire useful and important knowledge regarding the responses of this snow-sensitive species to future climate change. In addition, the specific objectives of this study were (1) to confirm the positive effect of the potential snow accumulation on the accuracy of the modelling process; (2) to define the realized niche model of *R*. *ferrugineum* in Andorra in order (3) to determine the habitat suitability of *R*. *ferrugineum* under different climate change scenarios.

## Materials and Methods

### Study area

The study was carried out in the Principality of Andorra (eastern Pyrenees), a mountainous country with an altitudinal range from 848 to 2,942 m a.s.l. that extends from 42°25’ to 42°39’ N and 1°24’ to 1°47’ E. The main climate type in Andorra is sub-continental with Mediterranean tendencies, although elevated areas of the territory enjoy a cold sub-oceanic climate [30]. Its singular and complex orography and its continentality modify the precipitation regimes in the Mediterranean area and the warm season is also the rainiest season of the year, as shown by the Climatological Atlas of Andorra [26]. Another particularity of the Andorran climate is the penetration of Atlantic air masses from the French side of the Pyrenees, which affect above all the high-altitude areas in the northern half of the Principality and guarantee a six-month snow season (November–April) on the country’s highest peaks [31].

These climatic particularities and its rugged topography guarantee great habitat diversity in the 468 km^2^ of the Principality, of which 248 km^2^ correspond to habitats of Community Interest [32] with a predominance of forests (181 km^2^), subalpine and alpine grasslands (143 km^2^), screes and rocky outcrops (74 km^2^) and shrublands (43 km^2^).

### Study species

*Rhododendron ferrugineum* L. (Ericaceae) is an evergreen shrub with well-branched trailing stems that grows to a height of 70 cm in silica-rich soils. Its leaves have been described as being possibly toxic [33], which would explain the lack of mechanical damage caused by livestock to this species.

*R*. *ferrugineum* is one of the most abundant shrubs in the Pyrenees and the European Alps where it has a large geographical range, above all in subalpine and alpine stages at 1500–2500 m a.s.l. [34]. It usually establishes itself on west- and northwest-facing slopes where it finds the most favourable environmental conditions, and tends to colonize extensively grazed mountain areas by outcompeting other species [35]. Its particular facility to colonize is principally due to its ability to reproduce by selfing, outcrossing or large-scale downslope vegetative spread. Thus, this shrub tends to naturally dominate in many subalpine and alpine grasslands in the Pyrenees.

The distribution map of *R*. *ferrugineum* in Andorra was obtained from colour 0.25-m resolution, geo-rectified aerial photographs taken in August 2012. The manually digitalized on-screen photo-interpretation of *R*. *ferrugineum* was completed in 2013. Given that the visual interpretation of aerial photos is not 100% accurate [36, 37], it was essential to determine the precision of the digitalized map. The map’s accuracy was assessed during summer 2013 by chosing at random 200 field-sampling points located above all at the plant’s lower and upper distribution limits in Andorra, of which 100 were where the species was present and 100 where it was absent from the map. Finally, the overall accuracy was calculated using Cohen's kappa coefficient of agreement [38, 39], which gave a value of χ = 0.955, very close to the value for perfect agreement (χ = 1).

Finally, to determine the realized niche model of *R*. *ferrugineum* in Andorra we used a dataset with 2000 randomly distributed presence/absence points (262 presence points, 1738 absence points, at least 200 m apart). Due to Andorra’s small size, and the wide distribution of *R*. *ferrugineum* within the country, 80% of the absence points are located less than 2.5 km from a *R*. *ferrugineum* presence point (1400 points), and almost all absence points less than 5 km from a presence point, which gives a more reliable representation of the species habitat suitability map, as suggested by [40].

### Climate data

The meteorological data for the modelling of the realized niche model of *R*. *ferrugineum* were obtained from the Climatological Atlas of Andorra [26], http://opengis.uab.es/wms/ACDA/index.htm), which provides high-resolution climate data for Andorra (90 x 90 m grid). High-resolution climate inputs are essential when SDMs are applied to mountain regions as complex topography can lead to uncertainties in the accuracy of species’ distribution [41]. We considered the annual mean minimal (Tmin) and maximal (Tmax) temperatures and the annual precipitation (Pann) according to the Atlas for the SDMs; the winter (December, January and February) minimum and maximum temperatures and the winter precipitation data were only used to compute the potential snow accumulation (Psnow) following [27] (Psnow map available in Appendix 1). We used Psnow to estimate snow cover duration since this variable can be implemented into climate change scenario modelling, and because physically based snow distribution models generate quite similar results to remote sensing data (i.e. the snow cover data obtained with SPOT images; [42]).

We expect that the SDM projection under climate changes scenario will be fairly accurate since the distribution of *R*. *ferrugineum* in Andorra is in equilibrium with climate and is little affected by land-use (in terms of species presence-absence) [43, 44]. Given that various climate change scenarios exist, all with inherent uncertainties and different prediction ranges [45], it is hard to determine which is the most realistic. Bearing in mind that it is important to determine the possible outcomes of worst- and best-case scenarios in the case of management and conservation planning decision-making, we used predictions from both the high and low ends of the range of scenarios (Intergovernmental Panel on Climate Change (IPCC)). We considered the A2, A1B and B1 IPCC emission scenarios that cover the entire range of scenarios: A2 lies close to the high end, A1B towards the middle and B1 close to the low end of the range. In terms of future climate projections for the Pyrenees ([46, 47]; SCAMPEI project: Climate Scenarios for Mountain Areas: Extreme Events, Snow Cover and Uncertainties, http://www.cnrm.meteo.fr/scampei/), warming trends are predicted for air temperatures but uncertainties appear in forecasts of precipitation. Studies on complex terrain such as Andorra also have to take into account the difficulties that projections face up to when attempting to capture the effects of the climate regimes that influence this area (Mediterranean, Continental and Temperate-Atlantic), as well as the local variability accentuated by the complexity of the relief. Elsewhere, inherent uncertainties are seen to exist in models. Once aware of these known limitations, regionalization or downscaling (dynamical or statistical, VALUE: COST Action ES1102 2012–2015 project, http://www.value-cost.eu/) is a good option for diminishing the problem of scale. Some of the previously cited scenarios work in this way, as do the high-resolution climate projection outputs derived from the model chain Arpege-Aladin (SCAMPEI project) chosen for our research. Due to the fact that the research is centred on Andorra, downscaled projections surrounding the Principality (four points) were adopted and averaged for precipitation (snow plus rain) and temperature (maximum and minimum) for the winter period and for the whole year for the reference periods 2021–2050 and 2071–2100. The derived values are shown in Appendix 2.

### Climatic and environmental data selection

In addition to the three available climatic variables (Tmin, Tmax and Pann) and the potential snow accumulation, four topographic variables were also considered in the modelization: elevation, slope, topographic aspect (cosine transformed) and solar radiation (obtained from the Andorra 10-m spatial resolution DEM).

Multicolinearity is frequent between environmental variables and can cause adverse effects, especially explication or estimation, which can lead to erroneous estimations of model predictions [48, 49]. The variables explaining over 60% of colinearity (*r*_pairwise_ ≥ 0.6) were removed from the analyses to give a more parsimonious model without collinear predictor variables [50]. Next, the multicolinearity between all the remaining explicative variables was tested with the variance inflation factor (VIF), [51, 48] in which the explicative variables with a value over 3 are removed from the analyses [52]. After checking for multicolinearity, only slope, topographic aspect, solar radiation and Psnow were retained.

### Spatial autocorrelation

Spatial autocorrelation due to the spatial structure of ecosystems, which cause special relationships between the elements that compose them [53], remains an important issue in the spatial prediction of species distribution [54] and can negatively affect the significance of correlation or regression coefficients between explicative and response variables [55, 56]. Our sampling methods did not overcome this problem [57] and by using Moran’s test [58] to check for spatial autocorrelation in our dataset we detected significant spatial autocorrelation (Moran’s I observed: 0.2449; Moran’s I expected: -0.0005; *P* < 0.001). In addition, we applied Spatial Eigenvector Mapping (SEVM) to add a new variable to the analysis. SEVM is one of the best methods for resolving the problem of spatial autocorrelation in species distribution models [59]. It is based on the extraction of eigenvectors from a connectivity matrix among spatial patterns, where the eigenvectors capture the spatial arrangement of the data points. Given that, each eigenvector represents a particular spatial patterning, the overall eigenvectors describe well the variation in space of the spatial autocorrelation. Eigenvectors permit the spatial arrangement of the data points to be translated into explanatory variables and to be used as predictors of the response variables. They also enable the spatial autocorrelation to be reduced notably [60, 59]. We obtained 13 eigenvectors from the SEVM computation, which were incorporated into the modelling process.

### Modelling framework

Since the existence of a single true model explaining species distribution is rarely justifiable in ecology [61, 62], the search for the best model must consider various modelling methods. The committee averaging method satisfies this necessity and consists of an ensemble forecasting method using different model algorithms that have varying levels of accuracy under different circumstances [63]. This approach is particularly relevant in the case of species habitat suitability maps [63, 64] since a combination of different approaches will always be more robust when faced with uncertainty. As well, given our objective of assessing the possible impact of climate change on *R*. *ferrugineum* suitability in Andorra, this approach permits a single consistent distribution model to be generated in a habitat suitability map and to be extrapolated for each climate scenario in both the short and long terms. To model the suitability of *R*. *ferrugineum* in Andorra we used five model algorithms available in the Biomod2 library (3.1–25, [65] in R 3.0.2, R Development Core Team, 2013): (1) the generalized additive model (GAM), a regression method [66]; (2) the multivariate adaptive regression splines (MARS), a mix of regression and classification methods [67]; (3) the classification tree analysis (CTA), a classification method [68]; (4) the boosted regression tree (BRT), a boosting algorithm [69] and (5) artificial neural networks (ANN), a machine-learning method [70]. GAM and MARS models are both generalized multiple regressions: GAM permits both linear and complex additive response patterns, as well as the combination of the two within the same model as smooth functions, while MARS uses linear regression, a mathematical construction of splines and binary recursive partitioning (rather than the smoother functions used in GAM) to perform a model with either linear or non-linear relationships between response and predictors [71 and reference therein]. The interest of these two methods is that they allow for the modelling of complex relationships between a response variable and its predictors [72]. For the classification and regression tree, the CTA involves rule-based methods that permit the capture of non-additive or complex interactions [71] and reference therein]. The BRT approach combines regression trees with gradient boosting, whereby an initial regression tree is fitted and iteratively improved (boosted) by minimising the variation in the response not explained by the model at each iteration [73]. Classification and regression trees are powerful tools for describing and predicting patterns of complex species distribution with environmental, physical and climatic variables and, moreover, provide coherent and interpretable results [74]. Finally, the ANN machine learning was used because it represents a robust method for modelling bioclimatic envelops that have non-linear responses to predictors [71] and reference therein]. Despite their respective advantages and disadvantages, these modelling approaches are highly useful and precise in the case of bioclimatic models of species distribution, in which the BRT, GAM and ANN methods provide particularly accurate models [73, 75].

For each modelling process, four cross-validation repetitions were performed and the data set was randomly split into the training set (70% of the initial data used for model calibration) and into the testing set (the remaining 30% used for model evaluation) to avoid underestimating the model’s accuracy [76]. Then, models were evaluated using the true skill statistic (TSS; [77]) and the area under the receiver operating characteristic curve (AUC; [78]). The AUC is a highly effective measure of the performance of ordinal score models and a threshold-independent measure of accuracy [29], while the TSS, a threshold-dependent measure of accuracy, has all of the advantages of Cohen's kappa statistic [79] but is not sensitive to prevalence [77]. The jackknife procedure that uses a ‘leave-one-out’ approach, commonly used in similar studies, was not implemented in this study because this method can generate over-optimistic estimates of predictive power with larger sample sizes [80]. TSS values ranged from– 1 to + 1, with– 1 corresponding to systematically wrong predictions and +1 to systematically correct predictions; TSS values > 0.6 are considered to be useful to excellent. AUC scores ranged from 0 to 1, with 0 for systematically wrong model predictions and 1 for systematically perfect model predictions; AUC values > 0.8 are considered to be good to excellent. Of the twenty models obtained, we only selected the models that exceeded the thresholds of TSS > 0.6 and AUC > 0.8 to compute the global model. Finally, the global model of the distribution of *R*. *ferrugineum* in Andorra was computed with the best performing models obtained and the *BIOMOD_Projection* function that allows for modelling under future climatic conditions.

### Decision threshold

We obtained the probability of the species habitat suitability using the global realized niche model of *R*. *ferrugineum*. However, in terms of the species sensibility to future climate change, information given in terms of presence/absence will be more practical than if presented as a probability. There are many subjective and objective approaches for determining the thresholds when defining ‘suitable’ and ‘unsuitable’ areas for the species [81]. While subjective approaches use highly arbitrary criteria, objective approaches are numerous and thresholds are chosen to maximize the agreement between observed and predicted distributions. The commonly used kappa maximization approach [81, 82] is not as good as the prevalence, average-predicted or sensitivity-specificity-combined approaches [81]. Following [81] and [83], we also used the mean of the threshold values to obtain two sensitivity-specificity-combined approaches: the sensitivity-specificity sum maximization approach [84, 83] and the sensitivity-specificity equality approach [84].

### The contribution of Psnow in the prediction of *R*. *ferrugineum* distribution

For the global realized niche model of *R*. *ferrugineum*, the importance of the four explanatory variables were calculated using the correlation between the global prediction and the prediction made with a randomized variable, where the importance of the variance was calculated as 1 –the correlation between models. The importance of variance was rescaled to between 0 and 1, where a high correlation between models was indicated by low influence and vice versa [85]. Finally, in order to assess the improvement when the potential snow accumulation was included in the prediction of the realized niche model, we also computed the modelling framework using Tmin, Tmax and Pann as climatic variables. The TSS and AUC scores of the models were used to compare the modelling processes with the TSS and AUC scores obtained considering the potential snow accumulation variable.

## Results

### Current habitat suitability map of *R*. *ferrugineum*

**Fig 1:**
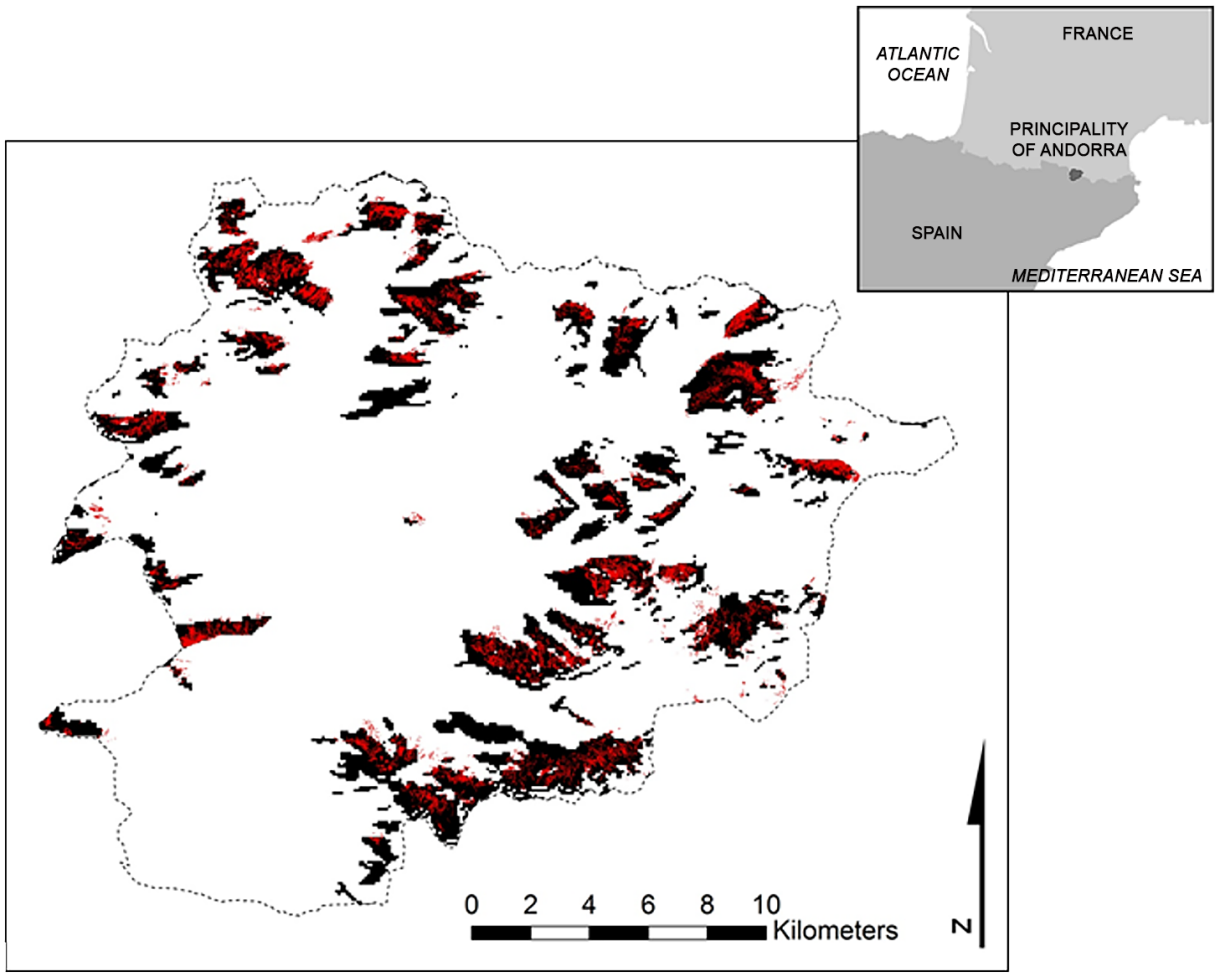
*Rhododendron ferrugineum* current potential distribution. The black tone indicates predicted areas of the current potential distribution of *Rhododendron ferrugineum* in Andorra taken from the global model at a resolution of 90 m.

Currently, *R*. *ferrugineum* covers about 14.66 km^2^ or 3.1% of the surface area of Andorra, above all (13.2 km^2^) between 2100 and 2500 m a.s.l (Fig 1). All five model algorithms used to predict its presence performed well. TSS values obtained were between 0.70 and 0.80 and AUC scores between 0.92 and 0.96 for the modelling process with the Psnow variable, while invariably lower TSS and AUC scores were obtained for the modelling process without other climatic variables (Table 1). These results provide a more than satisfactory prediction of a realized niche model of *R*. *ferrugineum* with the Psnow variable and so henceforth only this model will be considered for model projections under different climate change scenarios. In terms of the influence of the variables on species distribution in Andorra, Psnow is the most important variable (0.623); solar radiation too had a substantial impact (0.563), while the topographic aspect and slope had the least influence (0.077 and 0.068, respectively).

**Table 1:** Models AUC and TSS performance scores. Differences in the AUC and TSS performance scores in the five algorithms used to model the current distribution of *R*. *ferrugineum* in Andorra.

	Modelling process using Psnow as climatic variable		Modelling process usingTmin as climatic variable		Modelling process using Tmax as climatic variable		Modelling process using Pann as climatic variable	
Algorithm	AUC score	TSS score	AUC score	TSS score	AUC score	TSS score	AUC score	TSS score
GAM	0.939	0.742	0.920	0.928	0.725	0.708	0.918	0.698
BRT	0.959	0.801	0.946	0.952	0.786	0.749	0.947	0.755
CTA	0.920	0.755	0.853	0.869	0.649	0.626	0.874	0.647
ANN	0.925	0.703	0.917	0.696	0.931	0.729	0.916	0.703
MARS	0.930	0.717	0.910	0.918	0.700	0.655	0.904	0.648

The final model (obtained from 20 models and using 0.515 as the mean of the threshold values) yields a habitat suitability map for the species of 70.7 km^2^ or 15.1% of the surface area of Andorra. These 70.7 km^2^ of suitable area mainly correspond today to grassland and resinous forest habitats (17.7 and 20.1 km^2^ of the suitable area for *R*. *ferrugineum*, respectively) and, to a lesser extent, to shrubland habitats (15 km^2^). Finally, screes and rocky hillside habitats, probably the most difficult terrain on which to become established, represent 14.4 km^2^ or 20.3% of the suitable area.

For the realized niche model of *R*. *ferrugineum*, we obtained a habitat suitability map characterized by slopes with gradients of 10–35 degrees on north-facing slopes, with an annual precipitation of 1000–1350 mm (200–275 mm as snowfall during the winter season), an annual mean maximum temperature of 5.9–9.5° (0.1–3.0° during the winter season) and an annual mean minimum temperature of 3.9–5.6° (-6 to -4.5° during the winter season).

### Future habitat suitability maps of *R*. *ferrugineum* under climate change scenarios

**Fig 2:**
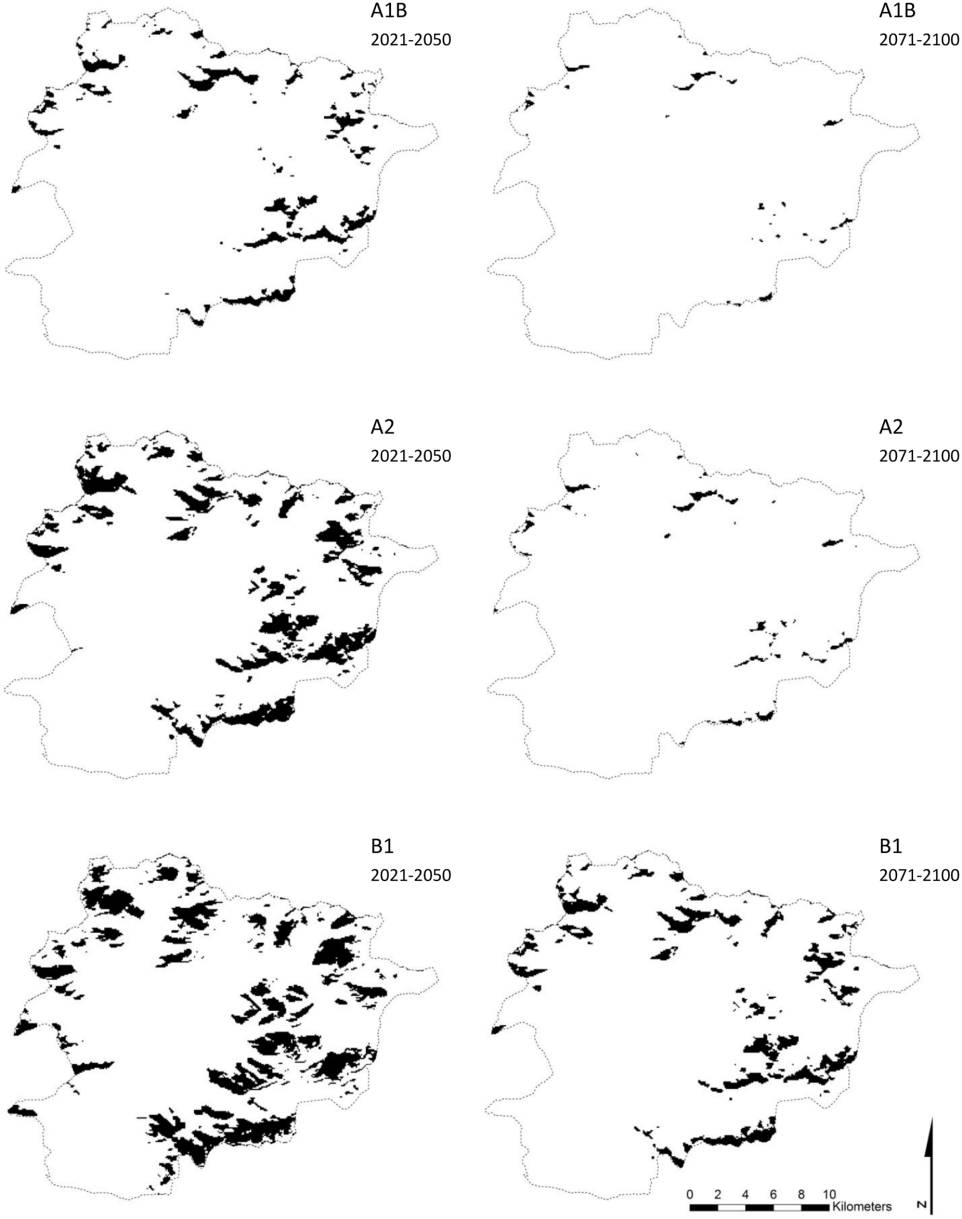
*Rhododendron ferrugineum* potential distribution under climate change. The black tone indicates predicted areas of the potential distribution of *Rhododendron ferrugineum* in Andorra under three climate changes scenarios (A1B, A2 and B1) for the periods 2021–2050 and 2071–2100 taken from the global model at a resolution of 90 m.

Using the mean of the two threshold values, we obtained from scenario A1B suitable habitat areas for *R*. *ferrugineum* of 22.2 km^2^ (4.7% of Andorra) and 2.8 km^2^ (0.6%) for the periods 2021–2050 and 2071–2100, respectively (Fig 2). In this case, 12.3% and 0% of the current suitable habitat area will be preserved for the species in the short and long terms, respectively. Currently, 6.1% of the short-term suitable habitat area is occupied by the species but none of the long-term suitable habitat area. For the A2 scenario, we obtained suitable habitat areas of 47.4 km^2^ (10.1% of Andorra) and 5.6 km^2^ (1.2%) in the short and long terms, respectively (Fig 2). In all, 42.6% and 0.2% of the current suitable habitat area will continue to be suitable habitat for the species in the short and long terms, respectively. In total, 13.9% and 0.6% of the area predicted as suitable habitat in the short and long terms, respectively, were occupied by the species in 2012. Finally, for the B1 scenario, we obtained suitable habitat areas of 68.6 km^2^ (14.7% of Andorra) and 32.8 km^2^ (7%) for the short and long terms, respectively (Fig 2), which means that 88.3% and 24.6% of the current habitat suitable areas will continue to be suitable habitat for the species in the short and long terms, respectively. In all, 16.1% and 10.3% of the area predicted as suitable habitat in the short and long terms, respectively, are already occupied by the species.

**Fig 3:**
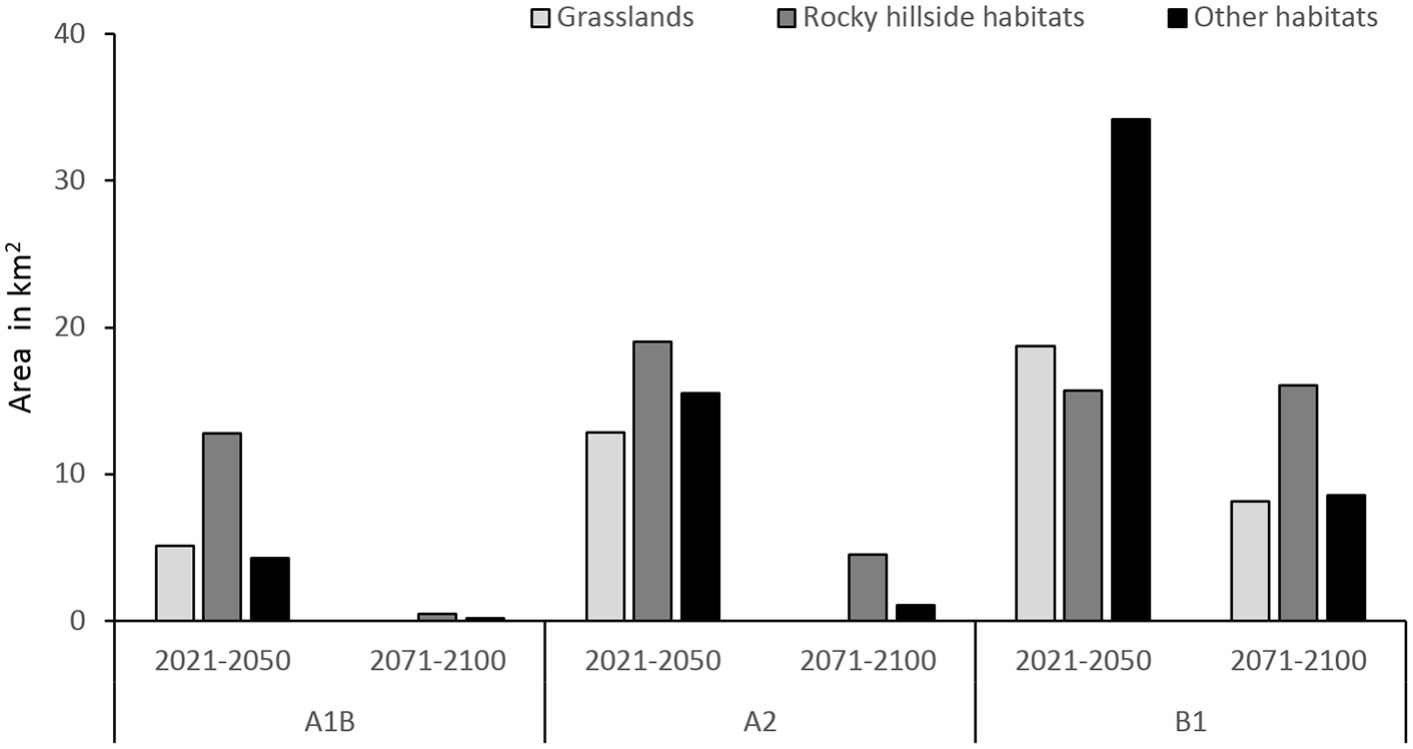
*Rhododendron ferrugineum* future suitable habitats under climate change. Future suitable habitats (grasslands, rocky hillsides and other habitats) present in the potential distribution of *Rhododendron ferrugineum* in Andorra under three climate change scenarios (A1B, A2 and B1) for the periods 2021–2050 and 2071–2100.

In the short term (2021–2050), the future suitable habitat area for *R*. *ferrugineum* will encompass, respectively, (a) grassland, and (b) screes and rocky hillside habitats as follows: 23% and 57.6% of the 22.2 km2 of suitable habitat areas predicted by the A1B scenario; 27.1% and 40.1% of the 47.4 km2 of suitable habitat areas predicted by the A2 scenario; and 27.3% and 22.9% of the 68.6 km2 of suitable habitat areas predicted by the B1 scenarios (Fig 3). In the long term (2071–2100), the future habitat suitable area for *R*. *ferrugineum* will cover above all screes and rocky hillside habitats: 84.4% of the 0.6 km^2^ and 81.3% of the 5.6 km^2^ of suitable habitat areas predicted by the A1B and A2 scenarios, respectively; while for the B1 scenario the future suitable habitat area of *R*. *ferrugineum* will encompass grasslands and screes and rocky hillside habitats: 24.9% and 48.9% of the 32.8 km^2^.

## Discussion

### Current habitat suitability map of *R*. *ferrugineum*

The excellent model accuracy obtained for the realized niche model of *R*. *ferrugineum* is not surprising since clonal species generally provide good model fits [12], especially when the modelled species—as is the case of *R*. *ferrugineum*–has a small geographical range [86]. This great accuracy can also be attributed to the relatively minor effect of land use on species presence-absence, the distribution of the species in equilibrium with climate (detected with the use of true absence data) and the niche stability highlighted by the species (no shift in competition with other species or genetic variation over time; [87, 88].

The use of potential snow accumulation to model the realized niche model of *R*. *ferrugineum* is of great interest as this variable had the greatest influence on species niche, thereby confirming the important role played by snow in its distribution. Our results also confirm those of [25, 42], whereby the variables that characterize limiting or regulating factors increase the predictive power of species niche modelling and, as in our case, contribute to greater model accuracy. Indeed, *R*. *ferrugineum* has a wide-ranging distribution within alpine environments because it is physiologically adapted to alpine habitats characterized by freezing, powerful solar radiation and sudden and important changes in microclimates and/or drought stress [89, 90]. Nevertheless, this species is highly dependent on snow cover during winter and so the consideration of potential snow accumulation provides a new dimension to the characterization of its realized niche. Given that solar radiation also contributes to species distribution, projections under different climate change scenarios will be numerous and thus permit good model accuracy [43, 44]. The lack of influence of slope on species niche implies that there will be little decrease in suitable conditions at higher elevations where slope gradients tend to be greater [91]. The limited influence of topographic aspect on the species’ distribution is initially surprising as the species tends to be located on north-facing slopes [92]. However, solar radiation highlights many variations in topographic aspect and as the prime input for energy balance also influences snow melting [93].

Although the altitudinal range of *R*. *ferrugineum* in Andorra is fairly similar to its observed range in the Alps [94] and other parts of the Pyrenees [95], we did find it in generally drier environments than on the northern face of the Pyrenees [92, 96] and in generally more humid environments than on the southern face [95]. Annual precipitation levels are not that important for this species—as other studies have already pointed out (see [97]; and reference therein)–and of far greater relevance is the effect that temperature has on precipitation in mountainous areas.

### Future habitat suitability maps of *R*. *ferrugineum* under climate change scenarios

The effect of climate change in the twenty-first century will include a reduction in the suitable habitat areas for *R*. *ferrugineum* in the Pyrenees. In the first half of this century, the decline in suitable habitats will be variable but not overly severe under the A1B, A2 and B1 scenarios (in the range 2.1–48.5 km2).The uncertainty existing regarding the magnitude of climate change gives rise to very contrasting forecasts for the species realized niche in this period. It is noteworthy that the moderate A1B scenario gives the most important reduction in suitable habitats for the species because the predicted climatic tendencies in winter under this scenario consist of a reduction in precipitation, while under the other scenarios an increase in the precipitation in winter is predicted. For the period 2071–2100, we obtained an important reduction in suitable habitat (in the range 37.9–70.1 km^2^), which is not surprising since snow cover, of crucial importance for the species presence, is directly related to climate [98] and will decrease in area in winter as a consequence of the increase in temperatures and the fall in precipitation (depending on the scenario) [23]. The upward shift of this species realized niche model will depend on the presence of snow but, given its slow colonization rate [99], it is possible that this shift may not actually occur.

Given that *R*. *ferrugineum* is a slow-growing species [100], the expected climate changes scenario may affect it particularly severely [101, 102], and its sensitivity to the climatic factors described above will provide a better understanding of its reactions. First of all, Mediterranean shrublands may benefit from future climate changes and occupy more suitable habitats in the Pyrenees [29], leading to species turnover and the replacement of *R*. *ferrugineum* at its lower limit by shrubland species from lower elevations such as *Buxus sempervirens* and *Corylus avellana*, or even by subalpine forests of *Abies alba* and *Pinus uncinata*. As well, the stabilizing effect that *R*. *ferrugineum* has on mountain soils will be fulfilled by other shrub species. Secondly, its plasticity and the frequency of its clonal reproduction [100] suggest that plants will be long-lived once they establish themselves. Thirdly, the vulnerability of seedlings will probably increase with the fall in precipitation predicted in scenarios A1B and A2: summer droughts like that of 2012 [103] may affect seedling survival [92] and are expected to increase in this southern part of the Pyrenean chain [104]. Fourthly, the consequences for plant survival and plant seed production may be even more serious if this species has to withstand winter and spring frosts if snow cover is not present (the mean minimum temperature during the winter season will range between -4.8 and -1°). Indeed, an important reduction in snow cover duration between at 1500–2000 m a.s.l. in the Pyrenees is expected under future climate change scenarios [105], which may expose *R*. *ferrugineum* to greater damage by freezing. Moreover, the greater resistance of subalpine and alpine grasslands to invasive species in the event of climate change and vegetation shifts [106] means that we should expect poorer seedling establishment and less and slower colonization of new areas. Besides, given that many of the new suitable habitats are situated on rocky hillside (23–84% of future predicted niches), the colonization of new areas will not be easy. The most likely outcome is therefore a shrinking of the current species distribution and a shift to the higher elevations where the few current areas of suitable habitats covered by the species will become the species main nucleus (from 7.3 to 0.5 km^2^ for the B1 and A2 scenarios in the long term, respectively, and none for the A1B scenario). Even so, it is still unclear whether this colonization will actually occur or not. Finally, a counterbalance to this negative outcome for the survival of *R*. *ferrugineum* may possibly be provided by the lower sensitivity of the snowpack (resulting from snow accumulation) to future climate change on the north-facing slopes [107] where the species is mostly found. Mountains are also relatively complex from a topographic and geomorphologic point of view; snow transport by wind is common and leads to snow erosion and/or accumulation depending on the fine scale of the wind circulation model [25]. In the case of a plant such as *R*. *ferrugineum* that is particularly dependent on the presence of snow cover, the redistribution of the snowpack under future climate change could create persistent snow patches at particular sites and raise the plant dispersal pattern, or reduce the lack of snow cover [25]. However, any such prediction would require higher resolution DEM data and a dominant wind model in the SDM models, neither of which are currently available for Andorra. Next, the lack of snow cover could even be partly offset by higher temperatures, which may result in less adverse conditions for the species since low temperatures are directly linked to vital physiological processes [108]. In this case, the more suitable conditions induced by climate change could allow the species to outcompete other shrubland or forest species and to persist wherever it becomes established.

## Conclusions

These data and techniques, combined with the potential snow accumulation, enabled us to obtain an accurate and realistic prediction of the realized niche model of *R*. *ferrugineum* in Andorra and the possible effects of climate change on its habitat suitability. A future challenge is to extend climate scenarios to other SDM models (thereby reducing the uncertainty) under climate change scenarios using other higher resolution models for the Pyrenees that include a model incorporating snow redistribution by wind. Here, despite these limitations, the introduction of the potential snow cover in the modelling framework gave a predicted important reduction in the species’ realized niche models by the end of the century, with possible important collateral consequences on black grouse populations whose principal habitat may be negatively affected. Finally, since *R*. *ferrugineum* is a relatively long-lived species [35] and may persist for a long time wherever it is established, decades may be needed to detect changes in its range [91, 109]. Moreover, the effect of changes in land use in Andorra and the ensuing reduction of livestock grazing in its subalpine and alpine grasslands [110] may affect the impact that climate change has on *R*. *ferrugineum* communities [111, 112] since species migration and disturbed species assemblages could lead to important shifts in the ecological interactions occurring within plant species communities [113]. Despite being very difficult to predict the effect of global warming on the distribution of upland species such as *R*. *ferrugineum*, the most important consequences of any future habitat reduction will be likely to concern black grouse conservation as this species represents a key habitat for this threatened Galliforme bird species.

## Supporting Information

S1 Fig Winter potential snow accumulation map. Winter potential snow accumulation calculated in Andorra following López-Moreno et al. (2007) using data from the Climatological Atlas of Andorra (Batalla et al. 2011). Dark blue tones indicate areas with high snow cover in winter and red areas indicate the current presence of the plant.
(TIF)

S1 Table Annual and winter Tmin, Tmax and precipitation values under climate change. Mean annual and winter (December, January and February) Tmin, Tmax and precipitation values for current reference values, for the mid-21st century (2021–2050) and for the end of the 21st century (2071–2100) under three climate change scenarios (A1B, A2 and B2).
(PDF)
