# Supplementary figures and images for: Modelization of the Current and Future Habitat Suitability of Rhododendron ferrugineum Using Potential Snow Accumulation
Source: PLoS One. 2016 Jan 29;11(1):e0147324. doi: 10.1371/journal.pone.0147324 (PMC4732742; doi:10.1371/journal.pone.0147324)

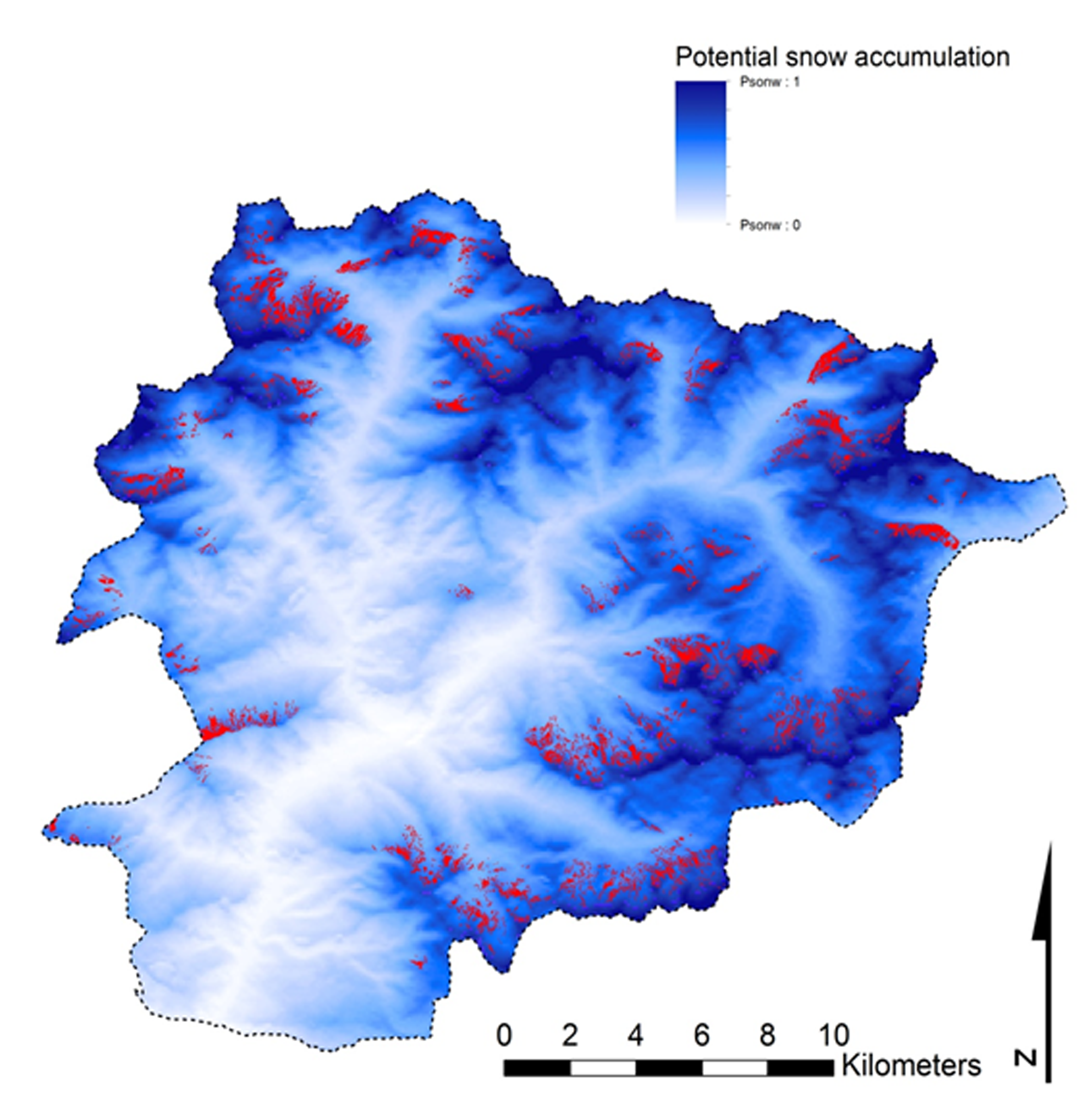

Supplement: S1 Fig — Winter potential snow accumulation calculated in Andorra following López-Moreno et al. (2007) using data from the Climatological Atlas of Andorra (Batalla et al. 2011). Dark blue tones indicate areas with high snow cover in winter and red areas indicate the current presence of the plant. (TIF) [file pone.0147324.s001.tif]
