# Supplementary material for: Modelization of the Current and Future Habitat Suitability of Rhododendron ferrugineum Using Potential Snow Accumulation
Source: PLoS One. 2016 Jan 29;11(1):e0147324. doi: 10.1371/journal.pone.0147324 (PMC4732742; doi:10.1371/journal.pone.0147324)
Supplement: S1 Table — Mean annual and winter (December, January and February) Tmin, Tmax and precipitation values for current reference values, for the mid-21st century (2021–2050) and for the end of the 21st century (2071–2100) under three climate change scenarios (A1B, A2 and B2). (PDF) [file pone.0147324.s002.pdf]

Scenario		A1B		A2		B1	
Period	Current	2021-2050	2071-2100	2021-2050	2071-2100	2021-2050	2071-2100
Annual Tmin (in ºC)	4.7	+ 1.4	+ 2.1	+ 1.0	+ 2.6	+ 1.1	+ 1.3
Annual Tmax (in ºC)	10.3	+1.9	+ 2.8	+ 1.3	+ 3.2	+ 1.2	+ 1.9
Annual P (in mm)	868.2	- 23.6	- 34.4	- 6.3	+ 15.2	+ 57.5	- 2.7
Winter Tmin (in ºC)	-1.1	+ 1.4	+ 2.7	+ 1.3	+ 3.5	+ 1.2	+ 1.8
Winter Tmax (in ºC)	3.5	+ 1.6	+ 3.7	+ 1.4	+ 4.6	+ 1.3	+ 2.4
Winter P (in mm)	288.0	- 35.2	- 160.1	+ 17.4	- 174.9	+ 50.2	- 62.8
